# Efficient electroporation in primary cells with PEDOT:PSS electrodes

**DOI:** 10.1126/sciadv.ado5042

**Published:** 2024-10-25

**Authors:** Asmaysinh A. Gharia, Clinton J. Bradfield, Elise P. W. Jenkins, Iain D. C. Fraser, George G. Malliaras

**Affiliations:** ^1^ Electrical Engineering Division, Department of Engineering, University of Cambridge, Cambridge, UK.; ^2^ Laboratory of Immune System Biology, National Institute of Allergy and Infectious Disease, National Institutes of Health, Bethesda, MD, USA.

## Abstract

Precise and efficient delivery of macromolecules into cells enhances basic biology research and therapeutic applications in cell therapies, drug delivery, and personalized medicine. While pulsed electric field electroporation effectively permeabilizes cell membranes to deliver payloads without the need for toxic chemical or viral transduction agents, conventional bulk electroporation devices face major challenges with cell viability and heterogeneity due to variations in fields generated across cells and electrochemistry at the electrode-electrolyte interface. Here, we introduce the use of microfabricated electrodes based on the conducting polymer poly(3,4-ethylenedioxythiophene) doped with polystyrene sulfonate (PEDOT:PSS), which substantially increases cell viability and transfection efficiency. As a proof of concept, we demonstrate the enhanced delivery of Cas9 protein, guide RNA, and plasmid DNA into cell lines and primary cells. This use of PEDOT:PSS enables rapid modification of difficult-to-transfect cell types to accelerate their study and use as therapeutic platforms.

## Teaser

PEDOT:PSS separates the delivery of electric fields from electrochemistry enabling high-efficiency cell transfection.

## INTRODUCTION

Electroporation is a well-established technique wherein cell membranes are transiently disrupted by pulsed electric fields to allow for the transport of otherwise impermeable molecules (*1*, *2*). While there are a variety of physical (*3*), chemical (*4*), and biological (*5*–*8*) methods to transfer biomolecules across the cell membrane, electroporation has remained a popular technique since the 1980s because of its ease of use, universal applicability, and reasonably high efficiency (*2*, *9*, *10*). Commercial electroporation devices apply pulsed electric fields with defined amplitude and duration in cuvettes or pipettes with embedded electrodes. These electrodes, often fabricated out of aluminum, stainless steel, platinum, gold, or graphite, are arranged as parallel plates to apply a uniform electric field across cells within the cuvette (*11*). While widely used, these bulk electroporation systems require high-voltage inputs and specialized capacitor discharge equipment to generate the required electric field intensity (*12*). In addition to high input voltage, bulk electroporation traditionally suffers from electric field distortion, heat generation, local pH variation, and metal ion dissolution, which negatively affect both electroporation efficiency and cell viability (*12*). To overcome these challenges, recent work has focused on miniaturization with microfluidic-based electroporators (*13*–*15*) as well as improving the electrode/electrolyte interface (*16*).

Microfluidic-based electroporation leverages the unique properties of microscale processing. Electrodes that are micrometers apart can generate required field intensities with low input voltage, provide uniform field distribution across individual cells, and rapidly dissipate heat all within a highly controlled fluidic and chemical environment (*17*, *18*), thereby addressing many of the drawbacks of bulk electroporation. Furthermore, low-volume microchannels enable the processing of individual cells to interrogate heterogeneity in cell populations, identify rare cells, and substantially reduce consumption of expensive reagents. These systems can be fabricated with additive manufacturing (*19*), conventional microfabrication techniques (*20*, *21*), or some combination thereof, while the use of transparent materials such as polydimethylsiloxane (PDMS) and glass further allows for real-time imaging to investigate electroporation processes at cellular resolution.

These advantages of microfluidic-based electroporation are realized further when microelectrodes are integrated with other fluidic processes such as cell sorting or hydrodynamic control (*22*, *23*) or other electronic processes such as electrophoresis, electroosmosis, or dielectrophoresis (DEP) (*24*, *25*). DEP has been used to substantially improve electroporation efficiency by precisely positioning cells between microelectrodes. DEP forces arise from nonuniform electric field gradients between particles and media with different dielectric properties. The magnitude of the force is proportional to the intensity of the electric field gradient, while the direction of the force can be tuned toward the higher electric field gradient [positive DEP (pDEP)] or toward the lower electric field gradient [negative DEP (nDEP)] as a function of frequency. Alternating pDEP and nDEP can thus be used to precisely pattern cells between electrodes and close to the surface in regions with high field uniformity. Cells randomly arranged between electrodes can also distort the local electric field and reduce the transmembrane potential in neighboring cells in what is known as the shadowing effect. This nonuniformity in induced transmembrane potentials results in heterogeneity across the cell population and reduces transfection efficiency (*26*, *27*). Precisely patterning cells between microelectrodes to position them within a locally uniform electric field and minimize shadowing can thereby reduce heterogeneity and improve transfection efficiency.

In addition to cell positioning, electrode composition greatly affects cell viability (*28*–*30*). At high voltages, metallic electrodes undergo electrochemical reactions at the electrode/electrolyte interface, inducing Faradaic currents. These irreversible Faradaic processes can produce gases, pH-changing species, chloride oxidation products, reactive oxygen species (ROS), and electrode dissolution (*31*–*33*). Commonly used electrode materials such as aluminum and stainless steel, alongside inert metals such as platinum, and gold can all induce these electrochemical reactions changing the local electrolyte composition (*34*–*36*). Conducting polymer electrodes have been shown to attenuate these irreversible electrochemical events in the context of electroporation (*16*). Poly(3,4-ethylenedioxythiophene) doped with polystyrene sulfonate (PEDOT:PSS) is one such conducting polymer that has been widely used in biomedical applications because of its biocompatibility, ease of processing, and reversible electrochemical properties (*37*–*39*).

In this study, we characterize electroporation efficiency and cell viability of PEDOT:PSS and uncoated metal microelectrodes in a microfluidic device. We find a range of optimal operating parameters by first using DEP pulses to pattern cells and then using biphasic pulses for electroporation. Contrary to previous reports (*16*), we find that PEDOT:PSS electrodes substantially improve cell viability and membrane permeabilization using fluorescent dyes. Using this approach, we further demonstrate that PEDOT:PSS electrodes can substantially improve electroporation efficiency while maintaining cell viability while transfecting biomolecules such as proteins, RNA, and DNA in both cell lines and primary cells.

## RESULTS

### Device design and fabrication

**Fig. 1.**
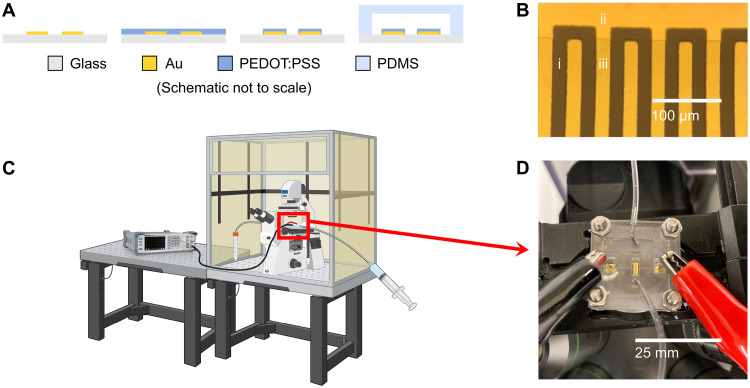
Illustration of system for PEDOT:PSS enhanced electroporation. (**A**) Simplified schematic of fabrication process wherein gold microelectrodes are photolithographically patterned onto a glass substrate. Multiple layers of PEDOT:PSS are deposited on the substrate and then etched to coat just the electrode digits. The electrodes are then enclosed in a microfluidic channel. (**B**) (i) Bare glass substrate, (ii) uncoated gold electrode, and (iii) PEDOT:PSS coating are visible on each 20-μm-wide and spaced electrode. (**C**) The entire device is placed in a fluorescence microscope with a CO_2_ and temperature-controlled environment [Created in BioRender. Fraser, I. (2024) BioRender.com/h54i032], and then (**D**) electrical and fluidic contact is established.

Our electroporation platform consists of planar interdigitated electrodes encased in a microfluidic channel visualized in Fig. 1. We pattern metal electrodes and the polymer coating (Fig. 1, A and B) using standard microfabrication techniques (fig. S1).

The electric field between electrodes gives rise to a transmembrane potential that disrupts membrane integrity and allows for the transport of impermeable chemicals and biomolecules. To study short-term cell viability and permeabilization, we stimulated cells on a fluorescence microscope (Fig. 1C) to quantify transport of fluorescent dyes.

**Fig. 2.**
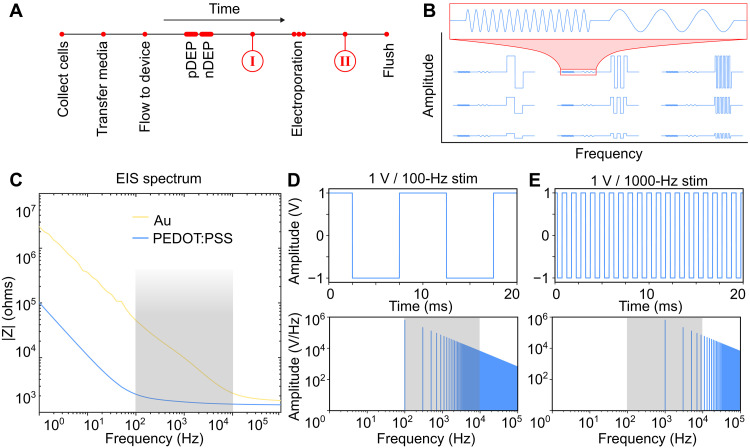
Workflow and stimulation parameters. (**A**) Timeline of the experiment wherein cells are transferred into electroporation buffer and injected into the device. pDEP is used to bring cells close to the surface, and nDEP is used to pattern cells between electrodes before the electroporation stimulation. Permeabilized cells are flushed out of the chip. Images of cells in the device at time points (I) and (II) are shown in Fig. 3A. (**B**) Schematic of tested signals wherein the pDEP and nDEP waveforms are unchanged and the amplitude and frequency of the electroporation pulses are varied. (**C**) Electrochemical impedance spectra. Gray area highlights the range between the gold and PEDOT:PSS cutoff frequencies. (**D**) Time domain (top) and frequency domain (bottom) of 100-Hz bipolar square wave pulse. (**E**) Time domain (top) and frequency domain (bottom) of 1000-Hz bipolar square wave pulse.

Figure 2 provides an overview of the device workflow (Fig. 2A) and stimulation sequences (Fig. 2B). First, we resuspended cells in a low-conductivity electroporation buffer with the desired transfection cargo. Cells were then injected into the microfluidic device and patterned between electrodes using DEP pulses. pDEP electrostatically polarizes cells relative to the surrounding media such that they are attracted toward electrodes and close to the device surface. nDEP polarizes cells such that they feel a repulsive force and are pushed between electrodes. Once uniformly patterned, cells were stimulated using biphasic pulses with a peak-to-peak voltage (Vpp) between 100 mV and 10 V and pulse frequencies ranging from 100 Hz to 100 kHz, giving rise to field intensities ranging from 50 V/cm to 5 kV/cm (Fig. 2B). Last, electroporated cells were pumped out of the microfluidic channel and plated to study long-term cell viability and permeabilization and characterize electroporation efficiency.

Electrochemical impedance spectroscopy (EIS) characterizes the electrode/electrolyte interface. Figure 2C is the Bode plot of devices with and without PEDOT:PSS coating in low-conductivity electroporation buffer. Here, we see that coated electrodes have a 10- to 100-fold reduction in impedance in the 100-Hz to 10-kHz electroporation stimulation frequency range. The principal frequency component of the bipolar square waves used in stimulation also falls within this range (Fig. 2, D and E).

### Fluorescent dye validation

**Fig. 3.**
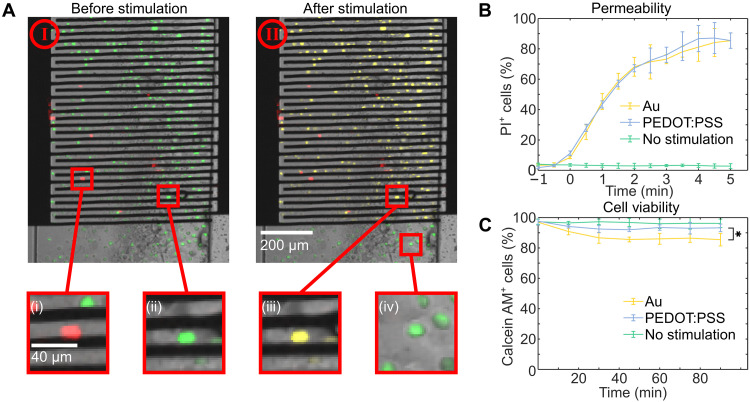
Time course of permeabilization and cell viability. (**A**) Cells patterned between electrodes. Dead cells express only PI (i), and living cells with undisrupted cell membranes express only calcein-AM (ii). After stimulation, cells between electrodes express both PI and calcein (iii); however, unstimulated cells do not express PI (iv). (**B**) The proportion of cells expressing PI in the 5 min following stimulation. (**C**) The proportion of cells expressing calcein-AM in the 90 min following stimulation The results are shown as the average across three Au (yellow) and three PEDOT:PSS (blue) devices with error bars for SD. *P < 0.05 (two-tailed Student’s *t* test).

We characterized short-term electroporation efficiency by stimulating cells while imaging in a fluorescence microscope in the presence of calcein-acetoxymethyl (calcein-AM) and propidium iodide (PI). Calcein-AM is a membrane permeable dye that is not fluorescent until the acetoxymethyl ester group is cleaved by intracellular esterases in living cells and converted into calcein. Calcein is then green fluorescent (ex, 494 nm; em, 517 nm) and is a marker for cell viability. PI, on the other hand, is impermeable to intact cell membranes. Once inside a cell, PI will intercalate nucleic acids and produce a strong red fluorescence (ex, 535 nm; em, 617 nm), thereby serving as a marker for membrane permeability. Figure 3A is an overlay of bright-field, green fluorescence and red fluorescence images of cells inside a microfluidic channel patterned between electrodes. Living cells with intact membranes are green because of the hydrolysis of calcein-AM, and dead cells are red because of intercellular PI and lack of esterase activity. Successfully electroporated living cells with disrupted membranes appear yellow because of the overlay of the green and red fluorescence signals. After DEP patterning but before the electroporation stimulation, >95% of visible cells were alive. After 400-ms stimulation with a 1-Vpp, 1-kHz bipolar square wave, all living cells that were between electrodes express both the calcein and PI signal, while living cells that were not between electrodes only express the calcein signal (see inset in Fig. 3A). This demonstrates that cells between electrodes were sufficiently disrupted during the electroporation stimulation to allow for the transport of PI across their membranes. Figure 3B shows the time course of the PI signal within cells stimulated with both uncoated Au and coated PEDOT:PSS electrodes. PI diffuses into cells soon after stimulation, and >80% cells express the dye within 4 min using either electrode type. To assess cell viability, we repeated the same stimulation conditions without PI, as the dye can be toxic to cells. We imaged cells on chip at 15-min intervals for 90 min after stimulation as seen in Fig. 3C. PEDOT:PSS-coated electrodes have significantly greater (*P* = 0.029 two-tailed Student’s *t* test) cell viability than gold electrodes with a 10% mean difference 90 min after stimulation. On-chip test conditions do not accurately represent the final use case as electroporation buffer is not an ideal culture medium and there was considerable variation in temperature and CO_2_ pressure in the microscope chamber. To minimize these effects, we performed long-term viability experiments with various stimulation conditions by flushing cells out of the device, changing the buffer to culture media, and culturing them under optimal conditions in an incubator at 37°C with 5% CO_2_ before measuring cell viability with the calcein assay. This protocol more closely resembles the conventional use of electroporation devices.

### Optimizing stimulation parameters

**Fig. 4.**
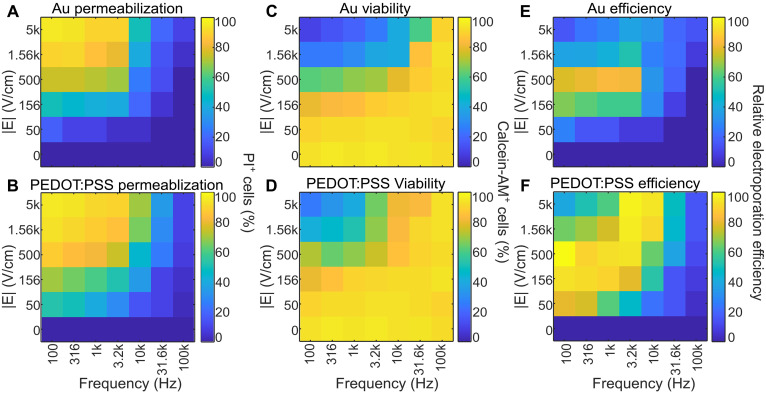
Optimization of operating parameters. (**A**) Permeabilization is quantified as the mean proportion of cells expressing PI on Au electrodes, or (**B**) PEDOT:PSS electrodes 4 min following stimulation on three devices. (**C**) Cell viability is characterized by the proportion of cells expressing calcein-AM on Au and (**D**) PEDOT:PSS electrodes 90 min after stimulation across three devices. (**E**) Pairwise multiplication of permeabilization and viability leads to an efficiency metric for Au and (**F**) PEDOT:PSS electrodes. Data are presented as a heatmap depicting the average proportion of fluorescent cells imaged on three devices.

We tested a matrix of operating conditions with the amplitudes and voltages with field intensities ranging from 50 to 5000 V/cm in the 20-μm region between electrodes. Frequencies below 100 Hz were not included in the analysis because of visible production of large gas bubbles on Au electrodes that greatly disrupted cells in the channel. Cells were imaged shortly after stimulation. Figure 4A (Au electrodes) and Fig. 4B (PEDOT:PSS electrodes) illustrate the proportion of cells between electrodes that express PI 4 min after electroporation at the various operating conditions. Low-frequency and high-amplitude stimulation tends to cause a greater proportion of cells to take up PI. With 5 kV/cm field and 100-Hz frequency, this results in a PI signal in 100% of cells on both Au and PEDOT:PSS electrodes. This is consistent with previous work (*20*, *25*) as membrane disruption is a function of both the transmembrane potential generated in an external electric field, as well as the duration of that stimulation. Larger amplitudes generate greater transmembrane potential, while lower frequency results in longer duration of that potential and ultimately more membrane disruption. The inverse also holds true; high frequencies and low-amplitude stimulation result in very few cells taking up PI. For example, 50 V/cm and 100-kHz stimulation results in ~6% of cells expressing a PI signal, while an average of 4% of unstimulated cells express a PI signal (*P* = 0.287). This nonsignificance indicates that a 50 V/cm field is not sufficient for electroporation. Au and PEDOT:PSS electrodes have very similar permeabilization efficiencies with field strengths greater than 500 V/cm and frequencies lower than 3.2 kHz. At lower field strengths (<500 V/cm) or higher frequencies (>3.2 kHz), coated electrodes exhibit greater permeabilization rates. For example, a 156 V/cm field at 100 Hz results in 44% permeabilization with gold and 59% permeabilization on PEDOT:PSS-coated electrodes (*P* = 0.0479). Similarly, a 1.56 kV/cm field at 10 kHz results in 39% permeabilization on gold and 62% on PEDOT:PSS electrodes (*P* = 0.0358).

Cell viability on the other hand tends to be low at high amplitudes and low frequencies. Figure 4 (C and D) demonstrates the proportion of cells with a calcein-AM signal 90 min after stimulation. A 5 kV/cm field at 100-Hz amplitude results in ~9% cell viability on Au electrodes and 17% viability on PEDOT:PSS electrodes with 6% SD. There was a significant difference in viability between Au and PEDOT:PSS electrodes at field strengths above 500 V/cm and frequencies below 10 kHz with PEDOT:PSS electrodes exhibiting up to 11% higher cell viability. At the other extreme, a 50 V/cm, 100-kHz field results in 95 and 96% viability on the Au and PEDOT:PSS electrodes respectively, an insignificant difference from the average 96% viability of unstimulated cells. This field intensity and frequency dependence of cell viability shares a mechanism with permeabilization efficiency wherein higher transmembrane potentials and durations result in greater membrane disruption and correspondingly greater cell death.

Optimizing electroporation efficiency requires maximizing membrane permeabilization while minimizing cell death. We chose to multiply the permeabilization efficiency and cell viability at different stimulation conditions to provide a simplified metric of electroporation efficiency for Au (Fig. 4E) and PEDOT:PSS (Fig. 4F) electrodes. As expected, electroporation efficiency is low in regions of low cell viability, such as when field intensities are high and frequencies low, as well as in regions where permeabilization is low such as when field intensities are low and frequencies high. Optimal electroporation occurs in the penumbral region where both permeabilization and viability are high, and it is these conditions in which PEDOT:PSS electrodes have two major advantages. The first is that PEDOT:PSS electrodes have both higher permeabilization and higher viability across all operating conditions, with up to 19% greater performance with our electroporation metric in conventional operating conditions (500 V/cm, 100 Hz) and up to 63% improvement in other operating conditions (5 kV/cm, 10 kHz). In addition to the bulk improvement in efficiency, another major advantage is the much broader range of operating conditions. A substantially higher proportion of the condition matrix for PEDOT:PSS shows efficiency readings of >80% (Fig. 4F, orange-yellow shading, compare to Fig. 4C). Optimal electroporation conditions are contingent on cell type (*11*) so improved electrode performance over a much broader range of stimulation parameters allows much greater scope for cell type–specific parameter optimization.

### Transfection validation

**Fig. 5.**
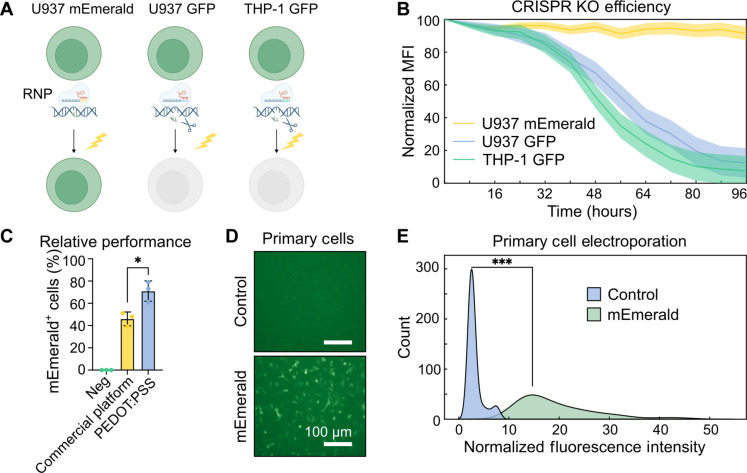
Validation in difficult-to-transfect cell types. (**A**) Schematic of RNP electroporation experiment in U937 and THP-1 cell lines. (**B**) Knockout efficiency following RNP transfection tracking the MFI of electroporated cells over 96 hours. (**C**) Relative transfection efficiency of mEmerald plasmid in THP-1 cells against Thermofisher Neon Transfection System 72 hours after stimulation. (**D**) Images of primary BMDMs 96 hours following mEmerald plasmid transfection. (**E**) Population distribution of fluorescence intensity in BMDMs 96 hours following mEmerald plasmid transfection. ***P < 0.001 (two-tailed Student’s *t* test).

To validate our electroporation performance, we evaluated transfection of ribonucleoprotein (RNP) complexes and plasmids into difficult-to-transfect cell types. THP-1^GFP+^, U937^GFP+^, and U937^mEmerald+^ human monocyte cell lines were transfected with Cas9 and green fluorescent protein (GFP) short guide RNA (sgRNA) RNPs from CRISPR-based knockout of GFP expression as illustrated in Fig. 5A. The mean fluorescence intensity (MFI) in GFP^+^ cells dropped 88 ± 12% within 96 hours of RNP electroporation, while mEmerald fluorescence dropped minimally in the same time (Fig. 5B). High U937^mEmerald+^ fluorescence also demonstrates high cell viability after transfection, while the significant reduction in THP-1^GFP+^ and U937^GFP+^ fluorescence suggests successful RNP transfection.

In addition to RNPs for CRISPR-based gene depletion, we transiently transfected THP-1 cells with an mEmerald-expressing plasmid and compared the PEDOT:PSS system performance to a commercial electroporation platform (Fig. 5C). Using the manufacturer’s recommended electroporation protocol, 46 ± 10% of cells expressed mEmerald with the Thermofisher Neon system, while 69 ± 9% of cells were fluorescent using the PEDOT:PSS platform 72 hours after transfection (*P* = 0.0202 two-tailed Student’s *t* test).

We quantified electroporation efficiency in primary cells by transfecting pTrix-mEmerald into murine bone marrow cells (BMCs) and differentiating the electroporated cells into bone marrow–derived macrophages (BMDMs). Figure 5D shows fluorescence in BMDMs 72 hours following transfection demonstrating both cell viability and high plasmid uptake. Single cells have a broad range in mEmerald expression with a significant (*P* = 0.00023) increase in fluorescence intensity relative to the median fluorescence of control cells stimulated without vector (Fig. 5E). THP-1 and U937 cells are 12 to 20 μm in diameter, while BMCs are 4 to 5 μm in diameter, demonstrating high transfection efficiency over a range of cell sizes.

## DISCUSSION

In this work, we demonstrate the efficacy of PEDOT:PSS microelectrodes for electroporation. The polymer coating reduces both impedance and electrochemical reactions at the electrode/electrolyte interface, allowing for cells to be electrically stimulated with minimal changes to their chemical environment (*40*, *41*). We used two-step dielectrophoretic positioning to pattern cells between electrodes in a uniform electric field generated between PEDOT:PSS electrodes in a microfluidic system. This allowed the full separation of field delivery from electrochemistry in a controlled environment and enabled a broader set of stimulation conditions. While large-magnitude and high-frequency electric fields can lead to high temperatures (*42*–*44*), thermal effects are assumed to be negligible for the electrode geometry and field parameters in this study (fig. S2). The identification of a broader set of operating conditions with PEDOT:PSS, achieving both high cell permeabilization and viability in typically intractable cells, is promising for optimizing genetic manipulation in essentially any cell type.

Increased permeabilization and reduced cell death can potentially be explained by electrochemical effects at the electrode/electrolyte interface. Previous work demonstrated that PEDOT:PSS coatings strongly attenuate the production of ROS and eliminate severe pH changes (*16*, *45*), both of which contribute to reduced cell viability. The EIS of Au and PEDOT:PSS electrodes in low-conductivity media illustrates the nature of the electrode/electrolyte interface and is shown in Fig. 2C. The resistive character of impedance exhibited by the PEDOT:PSS electrodes indicates minimal voltage loss at the electrode/electrolyte interface. This, in turn, generates a larger transmembrane voltage, which may explain improved membrane permeabilization in this frequency range.

Previous work has found that PEDOT:PSS-coated electrodes reduce membrane permeabilization (*45*). These studies explore the use of electrodes to stimulate cells in conductive media in context of in vivo stimulation. Permeabilizing cell membranes in conductive media requires charge injection and the generation of ROS. Hence, they leverage charge imbalanced waveforms to generate Faradaic currents. PEDOT:PSS coatings retain high charge balance during these stimulation pulses indicating limited electrochemistry. In the low-conductivity media that we use here, the electric field is not as strongly shielded by electric double-layer formation, suggesting that a transmembrane voltage can be sustained without a ROS-dependent mechanism. In this manner, PEDOT:PSS coatings improve permeabilization efficiency in low-conductivity media, while reducing efficiency at higher conductivity. In both instances, cell viability is improved. These results highlight the diverse applications of PEDOT:PSS for both in vitro electroporation and in vivo cell stimulation.

Aside from reversible electroporation to maximize gene transfer (*46*), irreversible electroporation has been used for pulsed field ablation (*47*, *48*). While PEDOT:PSS electrodes improve permeabilization efficiency across a range of stimulation conditions, the associated improvement in cell viability does not make them suitable for irreversible electroporation. This effect is apparent in Fig. 4 (E and F). Uncoated electrodes have low reversible electroporation efficiency but retain very high permeabilization efficiency with high-intensity, low-frequency fields. This region is smaller with PEDOT:PSS electrodes demonstrating reduced irreversible electroporation efficiency.

Further investigation is needed to determine the impact of PEDOT:PSS electrodes with cell types in more physiologically relevant media that could further reduce cell death. Additional modelling of field delivery in media with variable conductivity would benefit the choice of buffer for ideal cell stimulation (*49*). Aside from optimizing buffers, devices can be improved by leveraging sensing capabilities of PEDOT:PSS electrodes (*50*) to maximize electroporation efficiency. For example, the large surface area–to–volume ratio and low impedance of polymeric electrodes allows for high signal-to-noise measurements that would enable closed-loop feedback for single-cell individualized electroporation (*28*), with significantly lower costs due to the ease of thin film processing.

Future work would also benefit from measuring and studying the waveforms delivered to cells. In this study, while we initially verified DEP and electroporation pulses using an oscilloscope, we did not measure the full waveform during the experiment. It is possible that the delivered pulses were different from the generated signal. This limitation is mitigated in our study because both PEDOT:PSS and gold devices received the same generated waveform. However, recording and verifying the delivered pulses is especially important when studying different types of waveforms, such as nanosecond-pulsed fields. Recent advances in pulsed electric field stimulation demonstrate high-efficiency electroporation (*46*) and tissue ablation (*47*) with nanosecond-pulsed fields. Exploring nanosecond to tens of nanosecond pulses on our platform could further enhance permeabilization while minimizing potential heating and electrochemical effects. Future work on the role of PEDOT:PSS in electroporation devices will continue to enable new capabilities and reduce costs for a broad range of organic electronic devices.

## MATERIALS AND METHODS

### Electrodes

Interdigitated microelectrodes with 20-μm width and spacing were photolithographically patterned onto glass substrates. Photomasks (JD PhotoData, Hitchin, UK) were first designed using AutoCAD (AutoDesk, San Rafael, CA). Glass substrates were prepared by washing in isopropanol (IPA), acetone, and deionized water (DI H_2_O) and dehydrated on a hot plate at 150°C for 5 min. Then, photoresist (AZ nLOF2035, Microchemicals, Ulm, Germany) was spin coated on the substrate at 500 rpm for 5 s and 3000 rpm for 45 s and baked at 110°C for 60 s. After this, the substrate was loaded under the photomask in a mask aligner, exposed to i-line UV (105 mJ/cm^2^), and after exposure baked for 3 min at 110°C. The substrate was then developed in AZ726 MIF developer for 29 s until the microelectrode pattern was visible in the resist. The surface was then activated with oxygen plasma for 60 s, and then 5 nm of titanium and 100 nm of gold were deposited using E-beam evaporation (Kurt J Lesker Company, Jefferson Hills, PA). Photoresist was removed by a lift-off process in acetone for 15 min or until there is no longer ohmic contact measured between the electrodes.

The electrodes were coated with PEDOT:PSS using previously reported methods (*50*, *51*). Briefly, a PEDOT:PSS solution was prepared with a surfactant to improve film homogeneity and a cross-linker. This solution was spin coated on the electrodes at 500 rpm for 5 s and 3000 rpm for 30 s and baked for 60 s at 110°C. This spin coating was repeated a total of three times to produce a relatively thick (~575 nm) film before hard baking for 1 hour at 110°C. The hard baked devices were then soaked in DI H_2_O for 24 hours before lithographically patterning positive photoresist (AZ5214E, Microchemicals, Ulm, Germany) above the digits of the electrodes. Exposed PEDOT:PSS was removed using a reactive ion etch (Oxford Instruments, Abingdon, United Kingdom), and remaining photoresist was removed using acetone, leaving the final PEDOT:PSS microelectrodes.

### Microfluidics

A 150-μm-high, 1-mm-wide microfluidic channel was fabricated using conventional soft lithography (*52*). First, a thick negative photoresist (SU-8 2100, Kayaku Advanced Materials, Westborough, MA) was spin coated on a silicon substrate at 500 rpm for 10 s and 2000 rpm for 30 s. The edge bead was removed using propylene glycol monomethyl ether acetate (PGMEA, Sigma-Aldrich, St. Louis, MO), and film was baked at 65°C for 5 min and then 95°C for 20 min. Then, the film was aligned to a photomask, exposed with UV (240 mJ/cm^2^), and baked at 65°C for 5 min and 95°C for 10 min. Last, the film was developed in PGMEA with agitation for 10 min, rinsed with fresh PGMEA, followed by a second rinse with IPA, and air dried with nitrogen.

This mold was then placed in a vacuum chamber with 100 μl of Sigmacote (Sigma-Aldrich, St. Louis, MO) as a mold-release agent. PDMS (Sylgard 184, Dow Corning, Midland, MI) was prepared at a 10:1 base–to–cross-linker ratio, poured onto the mold, and baked at 80°C for 1 hour. The cured PDMS was peeled off the mold, diced, and had inlet and outlet holes punched. Both the microelectrodes and microfluidics were O_2_ plasma surface activated, the channel was aligned over the microelectrodes under a microscope, and the surfaces bonded.

### Packaging

Electrical connection pins (Ossila, Sheffield, United Kingdom) were clamped to contact pads at the edge of the glass substrate and epoxied in place to ensure a strong contact. A three-dimensionally printed holder was then screwed over the entire assembly for added robustness. Needles and tubing were attached to the inlet and outlet holes to establish fluidic contact, and a syringe was used to push media and cells through the channel.

### Electroporation buffer

We used a previously reported buffer (*25*) with 0.01× phosphate-buffered saline (PBS) and a conductivity of 0.03 S/m and supplemented with sucrose to an osmolarity of 180 mOsm/liter (*25*). One-part ribonuclease-free 1× PBS (Invitrogen, Carlsbad, CA) was added to 99 parts of DI H_2_O. Then, D(+) sucrose (60.58 g/liter) was added to adjust the osmolarity of the buffer to 180 mOsm/liter.

### Cell viability and permeability experiments

Devices were placed in a confocal microscope (TCS SP5; Leica, Wetzlar, Germany) to visualize cells during stimulation. Initially two fluorescent dyes, calcein-AM (final concentration of 5 μg/ml, Thermo Fisher Scientific) and PI (final concentration of 5 μg/ml, Thermo Fisher Scientific), were added to harvested cells to verify electroporation. In subsequent experiments, either PI was added to cells before to visualize membrane permeability or calcein-AM was added to visualize cell viability. Devices were sterilized by flowing through 1 ml of 70% ethanol in DI H_2_O and then rinsed with 1 ml of DI H_2_O. All electroporation experiments were done within 20 min of harvesting cells. In experiments to determine membrane permeability, PI dye (5 μg/ml final concentration) was added to cells immediately before stimulation, and a syringe (3 ml, BD, Franklin Lakes, NJ) was used to flow cells through the device. In experiments to determine cell viability, cells were stimulated on the device, flushed out, plated in a six-well plate, and cultured in an incubator for 48 hours. Cells were stained with calcein-AM 90 min before imaging.

### Stimulation parameters

Voltage was controlled by a 30-MHz arbitrary waveform generator (SDG1000X; RS Pro, Corby, UK) with sequential stimulation as follows:

1) pDEP: ac signal, 1 Vpp, 100 kHz, 5 s (*25*).

2) nDEP: ac signal, 1 Vpp, 10 kHz, 5 s (*25*).

3) Transfection: bipolar square wave with amplitude ranging from 0.1 to 10 Vpp and pulse width ranging from 100 to 100,000 Hz, 400 ms.

Five image fields were taken for each test condition. Images were processed with CellProfiler (*53*) to determine percentage of cells that expressed a fluorescent signal, and data were plotted using Python.

### Cell culture

U87 cells were cultured in Dulbecco’s modified Eagle’s medium (DMEM) (Gibco/Invitrogen), while THP-1 and U937 cells were cultured in RPMI 1640 (Gibco/Invitrogen), all media was supplemented with 10% fetal bovine serum (FBS) (Heat-Inactivated FBS, HyClone) and penicillin and streptomycin (100 U/ml) at 37°C and 5% CO_2_. Media was changed every 3 to 4 days with at a 1:5 cell-split ratio, and cells were harvested with 0.25% trypsin (Life Technologies). For electroporation, cells were spun down at 400*g* for 7 min and resuspended in electroporation buffer with a density of 1 × 10^6^ cells/ml.

### Primary cell

C57BL/6 background mice were maintained in an animal facility at the National Institutes of Health (NIH) under specific pathogen–free conditions, and all animal experiments were conducted in accordance with guidelines and regulations approved by National Institute of Allergy and Infectious Diseases Animal Care and Use Committee under protocol LISB 3E. BMCs were flushed from femur and tibia of 6- to 12-week-old animals and transfected with plasmid on our electroporation platform. They were then cultured in DMEM (Gibco/Invitrogen) supplemented with 10% FBS (HyClone), penicillin and streptomycin (100 U/ml), and macrophage colony-stimulating factor (50 ng/ml) at 37°C and 5% CO_2_.

### RNP electroporation

*Streptococcus pyogenes* Cas9 and sgRNA were ordered from Integrated DNA Technologies. sgRNA were designed to target mEmerald but not EGFP while maximizing specificity and minimizing off target effects with the following sequences: 5′-CGTAGGTCAAGGTGGTCACG-3′, 5′-CTGCACGCCGTAGGTCAAGG-3′, 5′-ACCCGCCACAACATCGAGGA-3′ 100 pmol of each sgRNA was added to 150 pmol of Cas9 in 5.4 μl of Tris-EDTA (TE) buffer and incubated for 20 min at room temperature to form the RNP complex. A total of 1 × 10^6^ cells in 1 ml of electroporation buffer were added to the RNP solution and pumped into the microfluidic chamber before stimulation pulses. Both THP-1 and U937 cells were stimulated with 3.16 V (1.56 kV/cm) at 3.2 kHz. Transfected cells were immediately pumped off chip into 20 ml of prewarmed complete RPMI 1640 and plated on 15-cm non–tissue culture–treated dishes.

### Plasmid electroporation

pTrix-mEmerald was prepared at a concentration of 500 ng/ml in DI H_2_O. THP-1 cells were transfected with either the Neon NxT Electroporation System (Thermo Fisher Scientific) with a 10-μl kit or on PEDOT:PSS-coated chips. Neon electroporation was conducted according to the manufacturer’s recommend protocol. Briefly, THP-1 cells were seeded at a density of 2 × 10^5^ cells/ml and then harvested and washed 3 days later in Dulbecco’s PBS without Ca^2+^ and Mg^2+^ (Gibco/Invitrogen). Cell were resuspended at a concentration of 2 × 10^7^ cells/ml in buffer R before being electroporated with 1 μg of plasmid in a 10-μl tip with voltage = 1700 V, pulse width = 20 ms, and pulse number = 1 (setting #5). Electroporated cells were immediately transferred into prewarmed complete RPMI 1640 without antibiotics and cultured a 37°C and 5% CO_2_ for 3 days before imaging. PEDOT:PSS electroporation was done with both THP-1 and BMC. Cells are harvested and resuspended in electroporation buffer at 1 × 10^6^ cells/ml concentration with 5 μg of plasmid. THP-1 cells were stimulated with 3.16 V (1.56 kV/cm) at 3.2 kHz, and hematopoietic stem cells were stimulated with 1 V (500 V/cm) at 10-kHz pulses.

### Statistical analysis

All significance testing was done using two-tailed Student’s *t* test using either Python or Prism (GraphPad). *P ≤ 0.05, ***P ≤ 0.001.

## Supplementary Materials

### This PDF file includes:

Figs. S1 and S2 Supplementary text References
