# Supplementary material for: Efficient electroporation in primary cells with PEDOT:PSS electrodes
Source: Sci Adv. 2024 Oct 25;10(43):eado5042. doi: 10.1126/sciadv.ado5042 (PMC11506140; doi:10.1126/sciadv.ado5042)
Supplement: Supplementary file 1 — Figs. S1 and S2 Supplementary text References [file sciadv.ado5042_sm.pdf]

Science Advances
AAAS

Supplementary Materials for

Efficient electroporation in primary cells with PEDOT:PSS electrodes

Asmaysinh A. Gharia *et al.*

Corresponding author: George G. Malliaras, gm603@cam.ac.uk; Iain D. C. Fraser, fraseri@niaid.nih.gov

Sci. Adv. **10**, eado5042 (2024)
DOI: 10.1126/sciadv.ado5042

This PDF file includes:

Figs. S1 and S2
Supplementary text
References

Fig. S1.

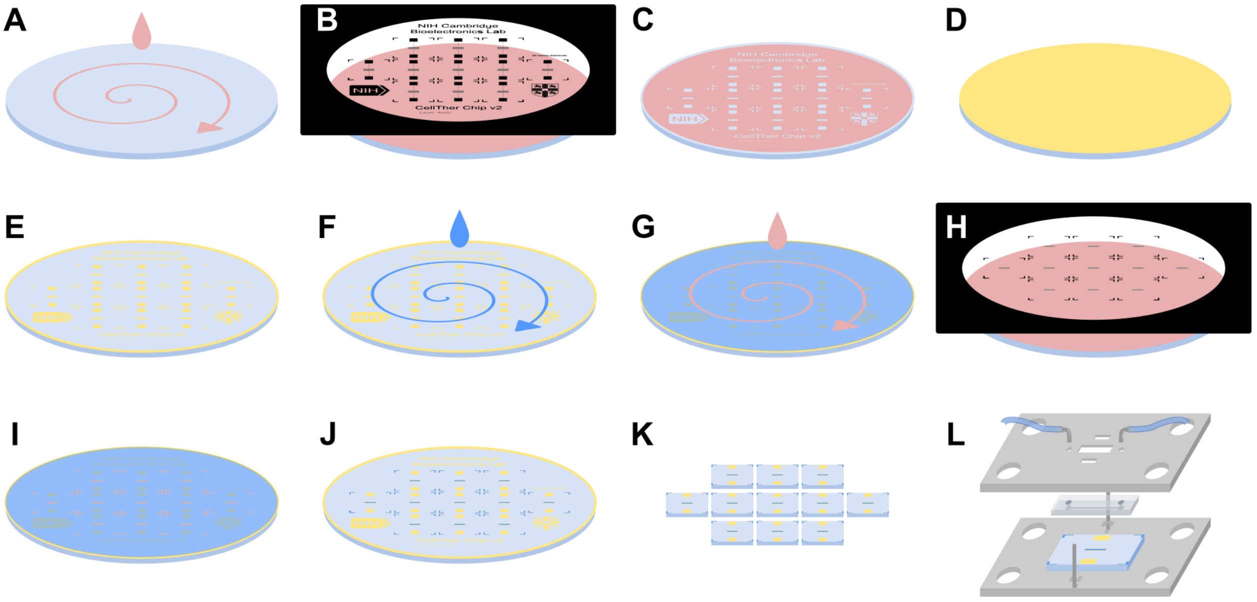

Microfabrication process of PEDOT:PSS coated electrodes. (A) Negative photoresist AZnLOF2035 is spin coated on a glass wafer and soft baked **(B)** patterned with a photomask and exposure to UV light **(C)** Un-exposed resist is removed in developer before **(D)** E-beam deposition of titanium and gold. **(E)** Exposed resist is lifted-off using acetone to reveal patterned metal electrodes. **(F)** PEDOT:PSS is spin coated onto the metal electrodes 3 times with a soft bake between spins to deposit a relatively thick polymer layer. **(G)** Positive photoresist AZ5214E is spin coated on the PEDOT:PSS coated wafer and **(H)** photolithographically patterned. Exposed resist is removed **(I)** in developer and **(J)** PEDOT:PSS removed with a reactive ion etch. Remaining photoresist is removed with acetone leaving a PEDOT:PSS coating over electrodes. **(K)** Devices are diced out of the glass wafer and **(L)** packaged with electric connector pins, microfluidics, a 3D printed holder, and fluidic tubing.

Fig.S2.

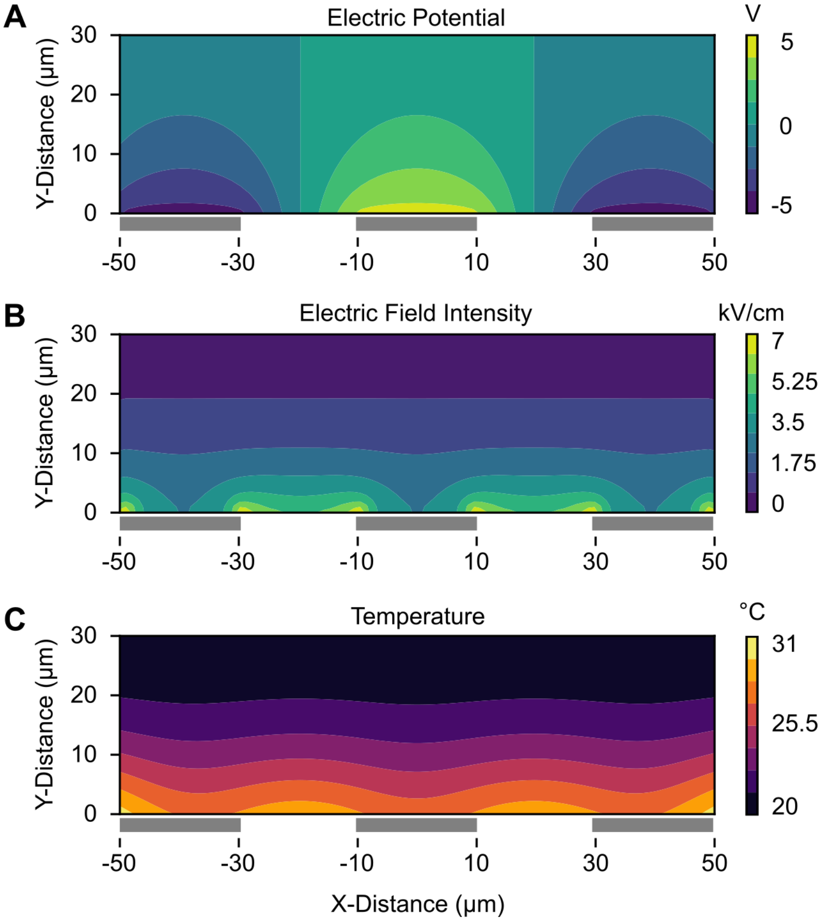

Simulation of electrical and thermal effects of interdigitated electrodes. (A) Analytical solution to electric potential around interdigitated co-planar electrodes with 20 um width and 20 um spacing and 10 Vpp stimulation. **(B)** Magnitude of the electric field as the gradient of the potential **(C)** Steady-state temperature due to Joule heating after dielectrophoretic cell capture and high-voltage, high-frequency square wave stimulation. Cells experience temperatures from 20-31°C due to Joule heating.

To model Joule heating, we first established an analytical model of the electric potential around interdigitated electrodes (*54*). Assuming infinitely long interdigitated electrodes, the potential can be written as

$$\phi(x, y) = \sum_{n=0}^{\infty} A_n \cos(k_n x)\, e^{-k_n y}$$

where

$$k_n = \frac{(2n + 1)\pi}{2d}$$

and d = 20μm. When electrode width and electrode spacing are the same, the Fourier coefficients are given by

$$A_n = \frac{16V_0}{\pi^2(2n+1)^2}\cos\left((2n+1)\frac{\pi}{4}\right)$$

Fig S2A illustrates a python simulation of this field potential around and above 3 interdigitated electrodes with 1 V stimulation. With the electric potential, we can calculate the electric field as

$$E = -\nabla\phi$$

The magnitude of the electric field |E| is simulated in Fig S2B. Finally, we can calculate the temperature distribution in the media due to the applied electric field as

$$\rho_m c_p\left(\frac{\partial T}{dt} + (u\cdot\nabla)T\right) = \nabla\cdot(k\nabla T) + \sigma|E|^2$$

where ρ_m is the fluid density, c_p is the fluid heat capacitance, T is temperature, u is the fluid velocity, k is fluid heat conductivity, and $\sigma|E|^2$ is the Joule heating term with σ conductivity and electric field. Assuming AC frequencies greater than 1 kHz, constant thermal conductivity, and neglecting thermal convection, the time averaged thermal distribution greatly simplifies (*42*) to

$$0 = k\nabla^2 T + \frac{1}{2}\sigma|E|^2$$

Figure S2C is a numerical solution to this equation using $k = 0.6\frac{W}{mK}$ and $\sigma = 0.03\frac{S}{m}$. The initial temperature distribution is assumed to be 20°C everywhere before stimulation with the electric field found in Fig S2B.

While we simulated Joule heating, localized dielectric losses were estimated from the model in (*43*) which was validated across a range of voltages and frequencies using an integrated temperature sensor directly between parallel electrodes. The temperature data and model in (*43*) both support less than $0.0152\frac{\Delta T}{V^2}$ temperature increase due to dielectric loss, even in low megahertz frequencies. The first 5 harmonics of the 5 kV/cm, 100 kHz square wave are all within this range and sum to a total temperature change less than 0.5°C.

Heat shock at temperatures greater than 42°C can impact cell viability (*55*). In our study we propose limited thermal impacts on cell viability because our normal device operations are unlikely to reach sufficiently high temperatures.

REFERENCES AND NOTES

1. I. Hapala, Breaking the barrier: Methods for reversible permeabilization of cellular membranes. *Crit. Rev. Biotechnol.* **17**, 105–122 (1997).

2. E. Neumann, M. Schaeferridder, Y. Wang, P. H. Hofschneider, Gene transfer into mouse lyoma cells by electroporation in high electric fields. *EMBO J.* **1**, 841–845 (1982).

3. E. G. Diacumakos, Methods for micromanipulation of human somatic cells in culture. *Methods Cell Biol.* **7**, 287–311 (1973).

4. F. L. Graham, A. J. van der Eb, A new technique for the assay of infectivity of human adenovirus 5 DNA. *Virology* **52**, 456–467 (1973).

5. D. H. Hamer, P. Leder, Expression of the chromosomal mouse Beta maj-globin gene cloned in SV40. *Nature* **281**, 35–40 (1979).

6. R. C. Mulligan, B. H. Howard, P. Berg, Synthesis of rabbit β-globin in cultured monkey kidney cells following infection with a SV40 β-globin recombinant genome. *Nature* **277**, 108–114 (1979).

7. M. L. Immordino, F. Dosio, L. Cattel, Stealth liposomes: Review of the basic science, rationale, and clinical applications, existing and potential. *Int. J. Nanomedicine* **1**, 297–315 (2006).

8. R. Fraley, S. Subramani, P. Berg, D. Papahadjopoulos, Introduction of liposome-encapsulated SV40 DNA into cells. *J. Biol. Chem.* **255**, 10431–10435 (1980).

9. J. C. Weaver, Y. A. Chizmadzhev, Theory of electroporation: A review. *Bioelectrochem. Bioenerg.* **41**, 135–160 (1996).

10. J. Shi, Y. Ma, J. Zhu, Y. Chen, Y. Sun, Y. Yao, Z. Yang, J. Xie, A review on electroporation-based intracellular delivery. *Molecules* **23**, 3044 (2018).

11. H. Potter, Electroporation in biology: Methods, applications, and instrumentation. *Anal. Biochem.* **174**, 361–373 (1988).

12. P. J. Canatella, J. F. Karr, J. A. Petros, M. R. Prausnitz, Quantitative study of electroporation-mediated molecular uptake and cell viability. *Biophys. J.* **80**, 755–764 (2001).

13. W. G. Lee, U. Demirci, A. Khademhosseini, Microscale electroporation: Challenges and perspectives for clinical applications. *Integr. Biol.* **1**, 242–251 (2009).

14. S. Movahed, D. Q. Li, Microfluidics cell electroporation. *Microfluid Nanofluid* **10**, 703–734 (2011).

15. T. Geng, C. Lu, Microfluidic electroporation for cellular analysis and delivery. *Lab Chip* **13**, 3803–3821 (2013).

16. G. Dijk, H. J. Ruigrok, R. P. O'Connor, PEDOT:PSS-coated stimulation electrodes attenuate irreversible electrochemical events and reduce cell electropermeabilization. *Adv. Mater. Interfaces* **8**, 2100214 (2021).

17. S. N. Wang, L. J. Lee, Micro-/nanofluidics based cell electroporation. *Biomicrofluidics* **7**, 011301 (2013).

18. N. Bhattacharjee, L. F. Horowitz, A. Folch, Continuous-flow multi-pulse electroporation at low DC voltages by microfluidic flipping of the voltage space topology. *Appl. Phys. Lett.* **109**, 163702 (2016).

19. S. Waheed, J. M. Cabot, N. P. Macdonald, T. Lewis, R. M. Guijt, B. Paull, M. C. Breadmore, 3D printed microfluidic devices: Enablers and barriers. *Lab Chip* **16**, 1993–2013 (2016).

20. A. Adamo, A. Arione, A. Sharei, K. F. Jensen, Flow-through comb electroporation device for delivery of macromolecules. *Anal. Chem.* **85**, 1637–1641 (2013).

21. H. Huang, Z. Wei, Y. Huang, D. Zhao, L. Zheng, T. Cai, M. Wu, W. Wang, X. Ding, Z. Zhou, Q. Du, Z. Li, Z. Liang, An efficient and high-throughput electroporation microchip applicable for siRNA delivery. *Lab Chip* **11**, 163–172 (2011).

22. T. Zhu, C. Luo, J. Huang, C. Xiong, Q. Ouyang, J. Fang, Electroporation based on hydrodynamic focusing of microfluidics with low dc voltage. *Biomed. Microdevices* **12**, 35–40 (2010).

23. J. Wang, Y. Zhan, V. M. Ugaz, C. Lu, Vortex-assisted DNA delivery. *Lab Chip* **10**, 2057–2061 (2010).

24. Z. Wei, X. Li, D. Zhao, H. Yan, Z. Hu, Z. Liang, Z. Li, Flow-through cell electroporation microchip integrating dielectrophoretic viable cell sorting. *Anal. Chem.* **86**, 10215–10222 (2014).

25. V. Jayasooriya, B. Ringwelski, G. Dorsam, D. Nawarathna, mRNA-based CAR T-cells manufactured by miniaturized two-step electroporation produce selective cytotoxicity toward target cancer cells. *Lab Chip* **21**, 3748–3761 (2021).

26. M. Pavlin, N. Pavselj, D. Miklavcic, Dependence of induced transmembrane potential on cell density, arrangement, and cell position inside a cell system. *IEEE Trans. Biomed. Eng.* **49**, 605–612 (2002).

27. R. Susil, D. Semrov, D. Miklavcic, Electric field-induced transmembrane potential depends on cell density and organization. *Electromagn. Biol. Med.* **17**, 391–399 (1998).

28. Z. Zhang, T. Zheng, R. Zhu, Single-cell individualized electroporation with real-time impedance monitoring using a microelectrode array chip. *Microsyst. Nanoeng.* **6**, 81 (2020).

29. H. G. Breunig, A. Uchugonova, A. Batista, K. König, Software-aided automatic laser optoporation and transfection of cells. *Sci. Rep.* **5**, 11185 (2015).

30. A. Fus-Kujawa, P. Prus, K. Bajdak-Rusinek, P. Teper, K. Gawron, A. Kowalczuk, A. L. Sieron, An overview of methods and tools for transfection of eukaryotic cells *in vitro*. *Front. Bioeng. Biotechnol.* **9**, 701031 (2021).

31. P. Turjanski, N. Olaiz, F. Maglietti, S. Michinski, C. Suárez, F. V. Molina, G. Marshall, The role of pH fronts in reversible electroporation. *PLOS ONE* **6**, e17303 (2011).

32. Y. Li, M. Wu, D. Zhao, Z. Wei, W. Zhong, X. Wang, Z. Liang, Z. Li, Electroporation on microchips: The harmful effects of pH changes and scaling down. *Sci. Rep.* **5**, 17817 (2015).

33. J. Mchardy, L. S. Robblee, J. M. Marston, S. B. Brummer, Electrical stimulation with pt electrodes. IV. Factors influencing pt dissolution in inorganic saline. *Biomaterials* **1**, 129–134 (1980).

34. G. Saulis, R. Lape, R. Praneviciute, D. Mickevicius, Changes of the solution pH due to exposure by high-voltage electric pulses. *Bioelectrochemistry* **67**, 101–108 (2005).

35. D. W. Kumsa, E. M. Hudak, N. Bhadra, J. T. Mortimer, Electron transfer processes occurring on platinum neural stimulating electrodes: Pulsing experiments for cathodic-first, charge-imbalanced, biphasic pulses for 0.566 □ k □ 2.3 in rat subcutaneous tissues. *J. Neural. Eng.* **16**, 026018 (2019).

36. A. Vizintin, J. Vidmar, J. Scancar, D. Miklavcic, Effect of interphase and interpulse delay in high-frequency irreversible electroporation pulses on cell survival, membrane permeabilization and electrode material release. *Bioelectrochemistry* **134**, 107523 (2020).

37. G. Dijk, A. L. Rutz, G. G. Malliaras, Stability of PEDOT:PSS-coated gold electrodes in cell culture conditions. *Adv. Mater. Technol.* **5**, 1900662 (2020).

38. M. J. Donahue, A. Sanchez-Sanchez, S. Inal, J. Qu, R. M. Owens, D. Mecerreyes, G. G. Malliaras, D. C. Martin, Tailoring PEDOT properties for applications in bioelectronics. *Mater. Sci. Eng. R Rep.* **140**, 100546 (2020).

39. S. Inal, J. Rivnay, A.-O. Suiu, G. G. Malliaras, I. McCulloch, Conjugated polymers in bioelectronics. *Acc. Chem. Res.* **51**, 1368–1376 (2018).

40. M. Berggren, G. G. Malliaras, How conducting polymer electrodes operate. *Science* **364**, 233–234 (2019).

41. E. Stavrinidou, P. Leleux, H. Rajaona, D. Khodagholy, J. Rivnay, M. Lindau, S. Sanaur, G. G. Malliaras, Direct measurement of ion mobility in a conducting polymer. *Adv. Mater.* **25**, 4488–4493 (2013).

42. S. J. Williams, N. G. Green, Electrothermal pumping with interdigitated electrodes and resistive heaters. *Electrophoresis* **36**, 1681–1689 (2015).

43. T. J. Kwak, I. Hossen, R. Bashir, W.-J. Chang, C. H. Lee, Localized dielectric loss heating in dielectrophoresis devices. *Sci. Rep.* **9**, 18977 (2019).

44. G. Anand, S. Safaripour, C. Snoeyink, Effects of frequency and joule heating on height rise between parallel electrodes with AC electric fields. *Langmuir* **38**, 1204–1214 (2022).

45. G. Dijk, H. J. Ruigrok, R. P. O'Connor, Influence of PEDOT:PSS coating thickness on the performance of stimulation electrodes. *Adv. Mater. Interfaces* **7**, 2000675 (2020).

46. A. Vizintin, S. Markovic, J. Scancar, D. Miklavcic, Electroporation with nanosecond pulses and bleomycin or cisplatin results in efficient cell kill and low metal release from electrodes. *Bioelectrochemistry* **140**, 107798 (2021).

47. E. Gudvangen, V. Kim, V. Novickij, F. Battista, A. G. Pakhomov, Electroporation and cell killing by milli- to nanosecond pulses and avoiding neuromuscular stimulation in cancer ablation. *Sci. Rep.* **12**, 1763 (2022).

48. M. C. Lefevre, G. Dijk, A. Kaszas, M. Baca, D. Moreau, R. P. O'Connor, Integrating flexible electronics for pulsed electric field delivery in a vascularized 3D glioblastoma model. *Npj Flex. Electron.* **5**, 19 (2021).

49. E. T. Jordan, M. Collins, J. Terefe, L. Ugozzoli, T. Rubio, Optimizing electroporation conditions in primary and other difficult-to-transfect cells. *J. Biomol. Tech.* **19**, 328–334 (2008).

50. S. Middya, V. F. Curto, A. Fernandez-Villegas, M. Robbins, J. Gurke, E. J. M. Moonen, G. S. Kaminski Schierle, G. G. Malliaras, Microelectrode arrays for simultaneous electrophysiology and advanced optical microscopy. *Adv. Sci.* **8**, 2004434 (2021).

51. J. Rivnay, P. Leleux, M. Ferro, M. Sessolo, A. Williamson, D. A. Koutsouras, D. Khodagholy, M. Ramuz, X. Strakosas, R. M. Owens, C. Benar, J.-M. Badier, C. Bernard, G. G. Malliaras, High-performance transistors for bioelectronics through tuning of channel thickness. *Sci. Adv.* **1**, e1400251 (2015).

52. J. C. McDonald, D. C. Duffy, J. R. Anderson, D. T. Chiu, H. Wu, O. J. Schueller, G. M. Whitesides, Fabrication of microfluidic systems in poly(dimethylsiloxane). *Electrophoresis* **21**, 27–40 (2000).

53. D. R. Stirling, M. J. Swain-Bowden, A. M. Lucas, A. E. Carpenter, B. A. Cimini, A. Goodman, CellProfiler 4: Improvements in speed, utility and usability. *BMC Bioinformatics* **22**, 433 (2021).

54. H. Morgan, N. G. Green, *AC Electrokinetics: Colloids and Nanoparticles* (Research Studies Press, 2003).

55. S. Lindquist, The heat-shock response. *Annu. Rev. Biochem.* **55**, 1151–1191 (1986).
